# Effects of a Natural Prolyl Oligopeptidase Inhibitor, Rosmarinic Acid, on Lipopolysaccharide-Induced Acute Lung Injury in Mice

**DOI:** 10.3390/molecules17033586

**Published:** 2012-03-22

**Authors:** Xiao Chu, Xinxin Ci, Jiakang He, Lanxiang Jiang, Miaomiao Wei, Qingjun Cao, Mingfeng Guan, Xianxing Xie, Xuming Deng, Jiakang He

**Affiliations:** 1 College of Animal Science and Technology, Guangxi University, 100 Daxue Road, Nanning 530005, Guangxi, China; 2 Key Laboratory of Zoonosis, Ministry of Education, Institute of Zoonosis, College of Animal Science and Veterinary Medicine, Jilin University, Changchun 130062, Jilin, China; 3 Department of Dermatology, Second Hospital of Jilin University, Changchun 130041, Jilin, China; 4 College of Plant Science, Jilin University, Changchun 130062, Jilin, China

**Keywords:** rosmarinic acid, LPS, SOD, MAPK, ALI

## Abstract

Rosmarinic acid (RA), a polyphenolic phytochemical, is a natural prolyl oligopeptidase inhibitor. In the present study, we found that RA exerted potent anti-inflammatory effects in *in vivo* models of acute lung injury (ALI) induced by lipopolysaccharide (LPS). Mice were pretreated with RA one hour before challenge with a dose of 0.5 mg/kg LPS. Twenty-four hours after LPS was given, bronchoalveolar lavage fluid (BALF) was obtained to measure pro-inflammatory mediator and total cell counts. RA significantly decreased the production of LPS-induced TNF-α, IL-6, and IL-1β compare with the LPS group. When pretreated with RA (5, 10, or 20 mg/kg) the lung wet-to-dry weight (W/D) ratio of the lung tissue and the number of total cells, neutrophils and macrophages in the BALF were decreased significantly. Furthermore, RA may enhance oxidase dimutase (SOD) activity during the inflammatory response to LPS-induced ALI. And we further demonstrated that RA exerts anti-inflammation effect *in vivo* models of ALI through suppresses ERK/MAPK signaling in a dose dependent manner. These studies have important implications for RA administration as a potential treatment for ALI.

## 1. Introduction

Acute lung injury (ALI) is a syndrome of acute inflammatory pulmonary edema, which has become a major public health concern [1]. Although the precise pathogenesis of ALI has not been fully elucidated, endotoxin or lipopolysaccharide (LPS) derived from Gram-negative bacteria play a critical role in ALI [2]. *In vivo* intratracheal administration of LPS has been extensively used as an experimental model of ALI or acute respiratory distress syndrome (ARDS), characterized by an acute inflammatory process in the airspaces and lung parenchyma [3,4]. The major pathological changes include the release of reactive oxygen species, pro-inflammatory cytokines, and chemotactic factors, which cause the aggregation of neutrophilic leukocytes and ultimately lung tissue injury [5,6]. The present study was designed to determine whether rosmarinic acid (RA) could ameliorate ALI induced by LPS in BALB/c mice.

RA is a polyphenolic phytochemical, which is found in many herbal plants including rosemary (*Rosmarinus officinalis*), oregano (*Origanum vulgare*) and mint (commonly *Mentha spicata*). It has a wide spectrum of biological effects, including anti-microbial, anti-inflammatory and immunomodulatory actions [7]. Moreover, RA inhibits LPS-induced production of COX-2 in raw264.7 mouse macrophages [8]. Our study was therefore aimed to investigate the influence of RA on LPS-induced ALI *in vivo*. Furthermore, in an attempt to study the possible mechanism of RA on LPS-induced ALI in mice, MAP kinase phosphorylation has been analyzed by Western blot.

## 2. Results and Discussion

### 2.1. Effects of RA on Cytokine Production from Mice with ALI

**Figure 1:**
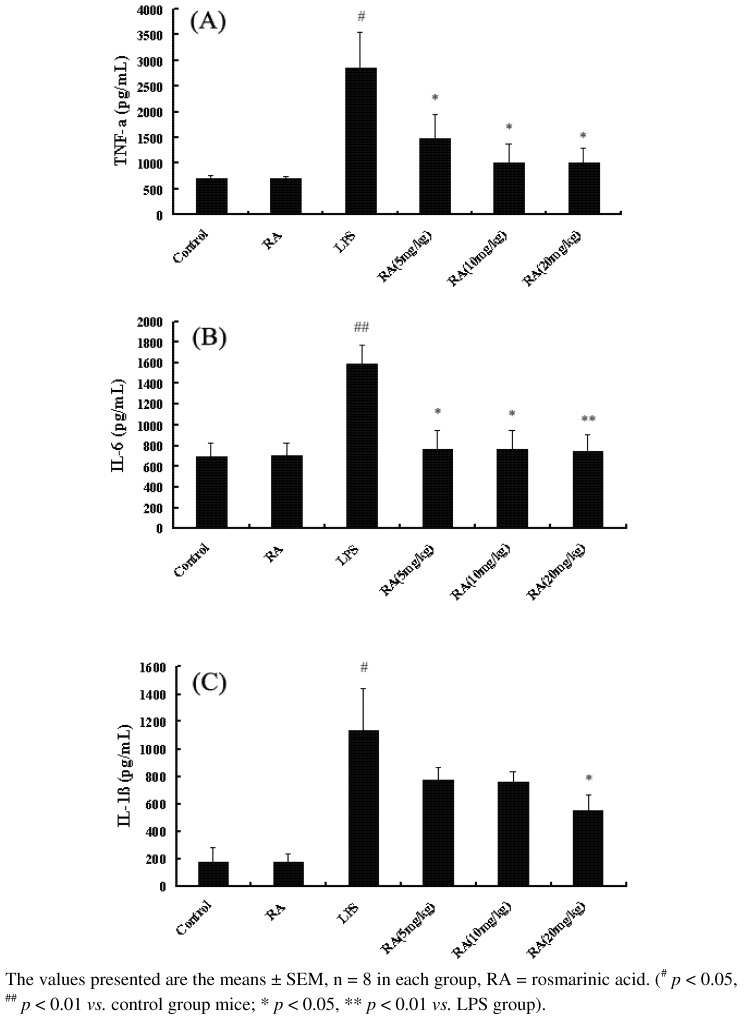
Effects of RA on TNF-α, IL-6, and IL-1β expression from mice with ALI. Mice were given RA (5 mg/kg, 10 mg/kg, and 20 mg/kg) by intraperitoneal injected 1 h before challenge with LPS. BALF was collected at 24 h following LPS challenge to analyze the inflammatory cytokines TNF-α (**A**), IL-6 (**B**), and IL-1β (**C**).

To investigate the role of RA on the production of cytokines *in vivo*, bronchoalveolar lavage fluid (BALF) was harvested 24 h after administering LPS for the analysis of cytokine concentration by ELISA. As shown in Figure 1, the level of TNF-α, IL-6, and IL-1β in LPS group increased significantly after LPS was given compared to those in the control group. RA (5 mg/kg) significantly decreased the production of LPS-induced TNF-α and IL-6 *in* *vivo*. However，only the highest concentration of RA (20 mg/kg) causes an inhibition of IL-1β.

### 2.2. Effects of RA on Inflammatory Cell Count in the BALF from Mice with ALI

**Figure 2:**
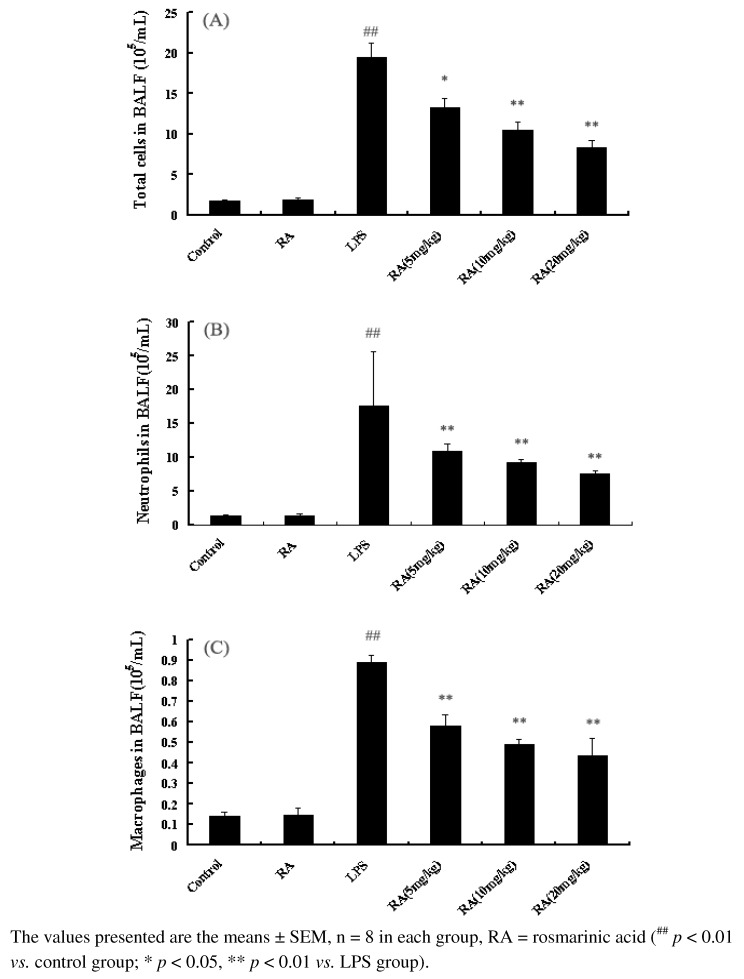
Effects of RA on the number of total cells, neutrophils, and macrophages in the BALF of LPS-induced ALI mice. Mice were given RA (5 mg/kg, 10 mg/kg, or 20 mg/kg) 1 h prior to an i.n. administration of LPS. BALF was collected at 24 h following LPS challenge to measure the number of total cells (**A**), neutrophils (**B**), and macrophages (**C**).

Mice exposed to LPS showed an increase in the total and differential cell counts in the BALF, as compared to the control group. As shown in Figure 2, RA (5, 10 or 20 mg/kg) treatment before LPS inhalation, led to a significant lowering of the number of total cells, neutrophils, and macrophages.

### 2.3. Effects of RA on Lung W/D Ratio in Mice with LPS-Induced ALI

**Figure 3:**
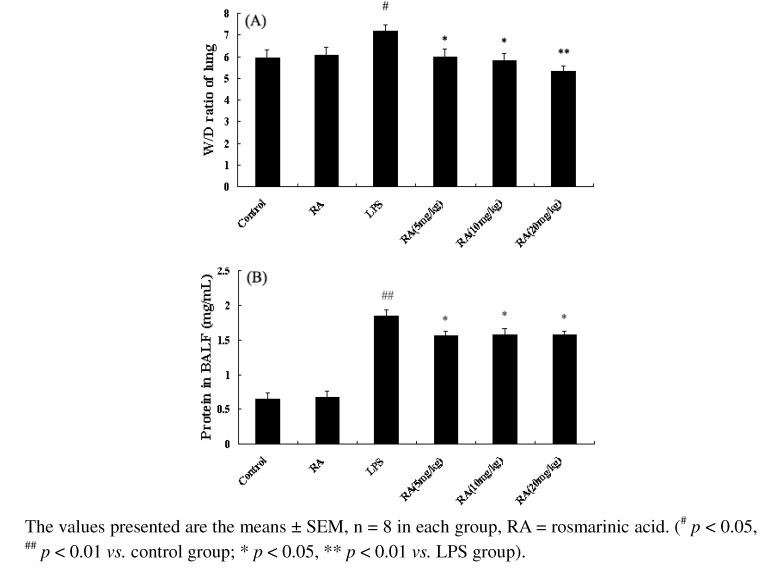
Effects of RA on the lung W/D ratio and total protein level in the BALF of LPS-induced ALI mice. Mice were given RA (5 mg/kg, 10 mg/kg, and 20 mg/kg) 1 h prior to an i.n. administration of LPS. The lung W/D ratio (**A**) and total protein concentration in the BALF (**B**) were determined 24 h after the LPS challenge.

Twenty-four hours after LPS was given, lungs were evaluated for edema by determining the lung water content. As shown in Figure 3A (LPS group), administration of LPS resulted in a significant increase in the W/D ratio compared to the control group (^#^ *p* < 0.05 *vs.* control group mice). RA significantly decreased the lung W/D ratio 24 h after LPS challenge in a dose dependent manner. As shown in Figure 3B, administration of LPS caused a significant increase in BALF protein levels 24 h after LPS instillation, compared with control mice. The protein content in the RA groups were significantly lower than the LPS group.

### 2.4. Effect of RA on SOD Activity from Mice with LPS-Induced ALI

**Figure 4:**
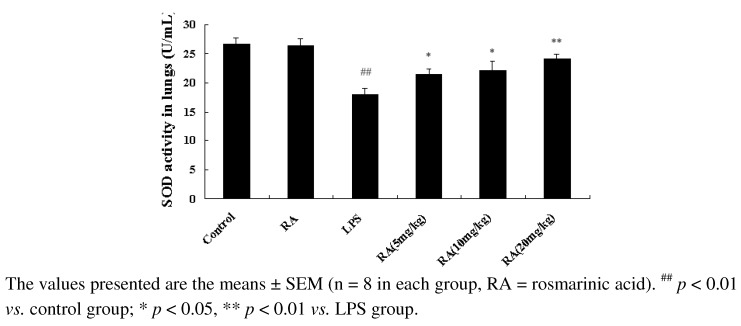
Effects of RA on SOD activity in BALF of LPS-induced mice. BALF was prepared from mice 24 h after LPS challenge. SOD activity was determined by SOD-specific ELISA kits.

Oxidative stress plays an important role in the development of LPS induced ALI [9]. To evaluate the effects of RA on oxidative stress, SOD activity in BALF was determined using a mouse SOD ELISA kit. As shown in Figure 4, LPS challenge resulted in significant decreases of SOD activity in the LPS group, compared with the control group. However, treatment with RA (5, 10 or 20 mg/kg) significantly increased SOD activity in LPS-induced BALF 

### 2.5. Effects of RA on Histological Changes in Lung Tissues from Mice with LPS-Induced ALI

**Figure 5:**
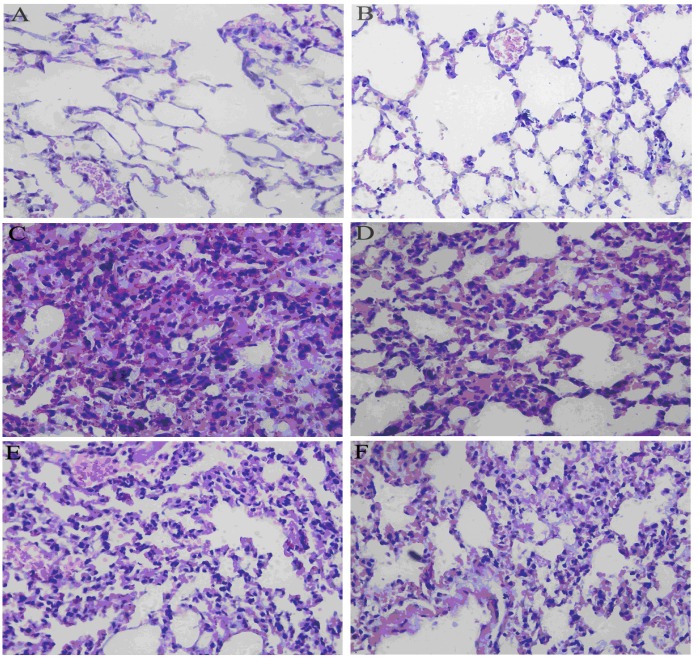
Effects of RA on histopathological changes in lung tissues in LPS-induced ALI mice. (**A**) PBS-challenged mice; (**B**) RA along treated mice (20 mg/kg); (**C**) LPS-challenged mice; (**D**) LPS-challenged mice treated with RA (5 mg/kg); (**E**) LPS-challenged mice treated with RA (10 mg/kg); (**F**) LPS-challenged mice treated with RA (20 mg/kg).

To evaluate the histological changes in lung tissue from mice with ALI following treatment with RA, lung sections obtained 24 h after administration of LPS were subjected to H&E staining. In the LPS group, the lung showed significant pathologic changes, such as alveolar wall thickening, alveolar hemorrhage, interstitial edema, inflammatory cells infiltration and even lung tissues destruction (Figure 5).

In addition, the control group also showed sight pathologic changes. However, treatment with RA (5, 10 or 20 mg/kg) significantly attenuated these changes (Figure 5).

### 2.6. Effects of RA on MAP Kinase Phosphorylation from Mice with LPS-Induced ALI

**Figure 6:**
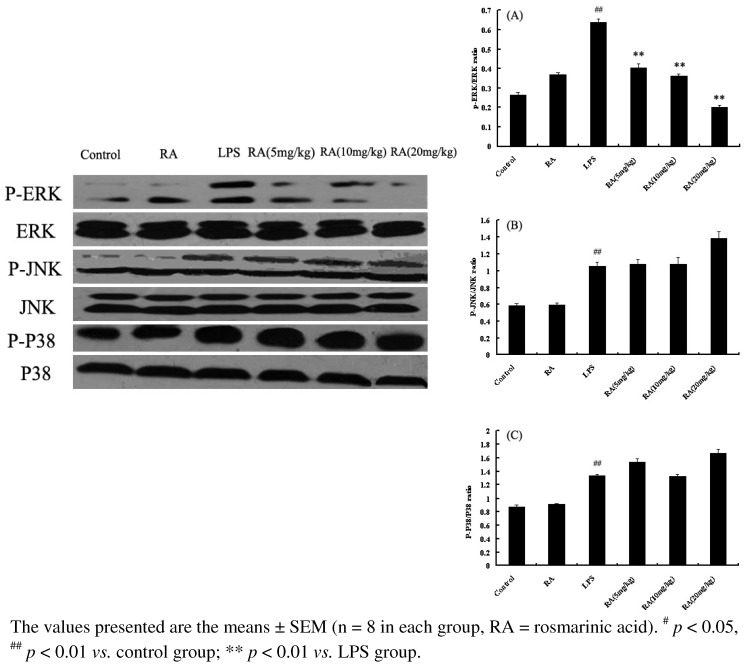
Effects of RA on MAPK activation *in vivo*. Total cellular proteins from lung were analyzed by Western blot with specific antibodies. Experiments were repeated three times and similar results were obtained.

Our data showed that LPS stimulation rapidly induced the phosphorylation of ERK, p38, and JNK in mice with LPS-induced ALI. RA (5, 10 or 20 mg/kg) significantly suppressed the ratio of p-ERK/ERK in a concentration-dependent manner (Figure 6). However, there was no significant change in the ratio of p-JNK/JNK and p-P38/P38 between LPS stimulation mice and the group treated with RA (Figure 6).

### 2.7. Discussion

Inflammation is critical in recruiting immune cells and molecules to the site of infection for defense. LPS, as a component of the cell walls of Gram-negative bacteria, has been recognized as a main component in the pulmonary inflammation and sepsis leading to ALI or ARDS [10,11]. Nowadays, the Traditional Chinese Medicine matrine has been shown to exhibit various anti-inflammatory properties [12,13]. RA is one active compound that partially accounts for the overall anti-inflammatory effect of *P. vulgaris* ethanol extracts. *P. vulgaris* ethanol extract significantly inhibited LPS-induced PGE2 and NO production [14]. Extracts from different accessions of *P. vulgaris* were screened for anti-inflammatory activity to identify accessions with the greatest activity [14]. To our knowledge, this is the first time to study the anti-inflammatory effect of RA in a nonspecific inflammation model in the lungs induced by LPS. Pro-inflammatory cytokines, TNF-α, IL-6, and IL-1β produced by macrophages in response to inflammatory stimuli and microbial products, play a critical role in ALI [15]. TNF-α is a primary mediator of the process of an inflammatory reaction [16]. TNF-α might originate from the neurons, from the myoblasts, or from monocaryon/macrophages [17], which both damage vascular endothelial cells, increasing their permeability, and furthermore induces alveolar epithelial cells to produce other cellular factors and chemotactic factors [18,19]. In patients with ALI and ARDS, elevated concentrations of IL-1β and TNF-α have been measured in BALF, and were related to poor outcome [20]. IL-6 is also a marker of the acute inflammatory cells response in the model of endotoxin-induced lung injury [21,22]. Inhibiting the overproduction of pro-inflammatory cytokines such as TNF-α, IL-1β, and IL-6 showed the lessening of pulmonary injury in LPS induced ALI model [23,24]. The data presented here demonstrates that RA may significantly inhibit the production of LPS-induced TNF-α, IL-6, and IL-1β *in vivo*. Giving TNF-α intravenously causes increased lung inflammation and increased permeability in animals [24,25,26,27] and neutrophil depletion prevented TNF-mediated acute lung injury in guinea pigs [28]. RA confers protection to mice ALI induced by LPS through the inhibition of the production of inflammatory cells in BALF. Moreover, excessive recruitment of polymorphonuclear leucocytes (PMNs) into the lung is a key event in the early development of ALI [29]. However, pretreatment with RA (5, 10 or 20 mg/kg) may inhibit inflammatory cell aggregation in the lungs. Consequently, RA confers protection to mice against ALI induced by LPS through the inhibition of the production of inflammatory cells and TNF-α, IL-6, and IL-1β.

Oxidative damage is a major cause of lung injury during ARDS. SOD is the only antioxidant enzyme that can scavenge superoxide and it has been reported to be markedly decreased in LPS-induced ALI [30]. The data presented here demonstrated that SOD was substantially increased in mice that were pre-administered RA, compared to the LPS group. Therefore, in this study we demonstrated that RA may prevent impairment of SOD activity during the inflammatory response to LPS-induced ALI.

Edema is a typical symptom of inflammation not only in systemic inflammation, but also in local inflammation. To quantify the magnitude of pulmonary edema, we evaluated the W/D ratio of the lung. Our experiments showed that RA may significantly inhibit edema of the lung, as shown by a W/D ratio in the RA group that was significantly lower than the LPS group.

MAPK molecules are among the important signaling pathways that control the synthesis and release of pro-inflammatory mediators by activated macrophages during the inflammatory response [31]. MAPKs contain three main families in mammalian species: p38 MAPK, ERK, and JNK. There are several reports that MAPKs play a pivotal role in the development of ALI [32,33]. In the present study, LPS stimulation led to evident phosphorylations of p38 MAPK, JNK, and ERK in mouse lung tissues. RA could repress the phosphorylations of ERK but not p38MAPK and JNK. It can be therefore inferred that RA down-regulated the expressions of inflammatory mediators through preventing the phosphorylations of MAPKs, mainly ERK.

## 3. Experimental

### 3.1. Animals

Male BALB/c mice, weighing approximately 18 to 20 g, were purchased from the Center of Experimental Animals of Baiqiuen Medical College of Jilin University (Jilin, China). The mice were housed in micro-isolator cages and received food and water *ad libitum*. The laboratory temperature was 24 ± 1 °C, and relative humidity was 40–80%. Mice were housed for 2–3 days to adapt them to the environment before experimentation. All animal experiments were performed in accordance with the guide for the Care and Use of Laboratory Animals published by the US National Institutes of Health.

### 3.2. Reagents

**Figure 7:**
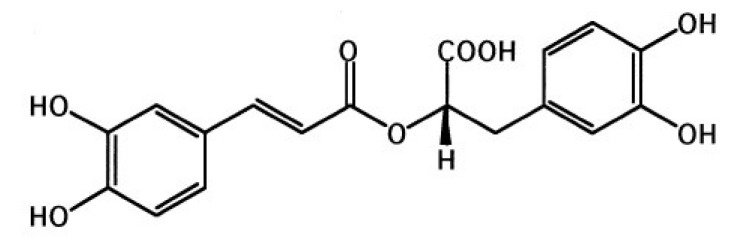
Chemical structure of RA.

RA (purity > 98%, Figure 7) used in the experiments was obtained as a lyophilized powder in a 20 mg vial manufactured by National Institute for the Control of Pharmaceutical and Biological Products (Beijing, China). LPS (from *Escherichia coli* 0111: B4) was purchased from Sigma-Aldrich (St. Louis, MO, USA). Mouse TNF-α, IL-6, and IL-1β ELISA kits were purchased from BioLegend (San Diego, CA, USA). Phospho-specific antibodies for ERK1/2, p38 and JNK as well as antibodies against ERK, p38 and JNK proteins were obtained from Cell Signaling Technologies (Beverly, MA, USA). Anti-rabbit and anti-mouse horseradish peroxidase (HRP)-conjugated secondary antibodies were purchased from PTG (Chicago, IL, USA). Mouse Super SOD ELISA kits were purchased from Uscn life Co. (Missouri City, TX, USA). All other chemicals were of reagent grade.

### 3.3. LPS-Induced ALI Model

Animals were divided into six groups each having eight animals. These groups were: (a) control, (b) LPS-treated, (c) RA treated (20 mg/kg), (d) LPS+RA (5 mg/kg) treated, (e) LPS+RA (10 mg/kg) treated and (f) LPS+RA (20 mg/kg) treated group. After the BALB/c mice were diethyl ether-anaesthetized, 10 μg LPS was instilled intranasally (i.n.) in 50 μL PBS to induce lung injury. Control mice were given 50 μL PBS i.n without LPS. RA (at doses of 5, 10 and 20 mg/kg, respectively) or vehicle (PBS) was intraperitoneal injected 1 h prior to LPS administration. Previous reports have shown that LPS can cause marked pulmonary inflammation as an acute injury after 2–4 h and maximizes at 24–48 h [34]. So in this study, BALF and tissue samples were collected 24 h after LPS exposure. Collection of BALF was performed three times through a tracheal cannula with 0.5 mL of autoclaved PBS, instilled up to a total volume of 1.3 mL. The fluid recovered from each sample was centrifuged (4 °C, 3,000 rpm, 10 min) to pellet the cells. The cell pellets were resuspended in PBS for total cell counts using a hemacytometer, and cytospins were prepared for differential cell counts by staining with the Wright-Giemsa staining method. At least 200 cells were counted per slide. 

### 3.4. Assays for Cytokines and SOD

The concentrations of cytokine TNF-α, IL-6, and IL-1β in the supernatants of the BALF were measured by sandwich enzyme-linked immunosorbent assay (ELISA) using commercially available reagents according to the manufacturer’s instructions. SOD activity in the BALF was quantified by using a mouse SOD ELISA kit.

### 3.5. Lung Wet-to-Dry Weight (W/D) Ratio

After mice were euthanized, the lungs were excised. Each lung was blotted dry, weighed, and then placed in an oven at 80 °C for 48 h to obtain the “dry” weight. The ratio of the wet lung to the dry lung was calculated to assess tissue edema.

### 3.6. Protein Analysis

Protein concentrations in the supernatant of the BALF were quantified using the bicinchoninic acid (BCA) method to evaluate vascular permeability in the airways.

### 3.7. Histopathologic Evaluation

Histopathologic evaluation was performed on mice that were not subjected to BALF. The lung tissue was fixed in formalin, dehydrated, paraffin embedded and sliced. After hematoxylin and eosin staining, pathological changes in the lung tissues were observed under light microscope.

### 3.8. Western Blot Analysis

Tissues were harvested and frozen in liquid nitrogen immediately until homogenization. Samples were homogenized in RIPA buffer and lysed for 30 min on ice. Total protein fractionation was performed using a cell lysis buffer for western blot and IP (Beyotime Institute of Biotechnology; China) according to the manufacturer’s protocol. Protein concentration was assayed using the Bio-Rad protein kit, and equal amounts of protein were loaded into wells on a 10% sodium dodecyl sulphate (SDS)-polyacrylamide gel. Subsequently, proteins were transferred onto polyvinylidene difluoride (PVDF) membrane, blocked overnight with 5% (wt/vol) nonfat dry milk, and probed according to the method described by Towbin *et al*. [35], with specific antibodies against JNK, ERK1/2, p38 and phospho-specific antibodies to JNK, ERK1/2 and p38 proteins in 5% (wt/vol) BSA dissolved in TTBS. With the use of a peroxidase-conjugated secondary anti-mouse or anti-rabbit antibody, bound antibodies were detected by ECL plus (GE Healthcare, Buckinghamshire, UK).

### 3.9. Statistical Analysis

All data were presented as the means ± standard error of the mean (SEM). Independent groups were compared using Mann-Whitney’s U-test. Statistical analysis was performed using the Kruskal-Wallis test. A *p* value of <0.05 was considered to be statistically significant.

## 4. Conclusions

In the present study, we found that RA exerted potent anti-inflammatory effects in *in vivo* models of lung injury induced by LPS. The efficacy may be due to inhibition of ERK/MAPK phosphorylation.
